# Inference of Expanded Lrp-Like Feast/Famine Transcription Factor Targets in a Non-Model Organism Using Protein Structure-Based Prediction

**DOI:** 10.1371/journal.pone.0107863

**Published:** 2014-09-25

**Authors:** Justin Ashworth, Christopher L. Plaisier, Fang Yin Lo, David J. Reiss, Nitin S. Baliga

**Affiliations:** 1 Institute for Systems Biology, Seattle, Washington, United States of America; 2 Department of Microbiology, University of Washington, Seattle, Washington, United States of America; The University of North Carolina at Charlotte, United States of America

## Abstract

Widespread microbial genome sequencing presents an opportunity to understand the gene regulatory networks of non-model organisms. This requires knowledge of the binding sites for transcription factors whose DNA-binding properties are unknown or difficult to infer. We adapted a protein structure-based method to predict the specificities and putative regulons of homologous transcription factors across diverse species. As a proof-of-concept we predicted the specificities and transcriptional target genes of divergent archaeal feast/famine regulatory proteins, several of which are encoded in the genome of *Halobacterium salinarum*. This was validated by comparison to experimentally determined specificities for transcription factors in distantly related extremophiles, chromatin immunoprecipitation experiments, and *cis*-regulatory sequence conservation across eighteen related species of halobacteria. Through this analysis we were able to infer that *Halobacterium salinarum* employs a divergent local *trans*-regulatory strategy to regulate genes (*carA* and *carB*) involved in arginine and pyrimidine metabolism, whereas *Escherichia coli* employs an operon. The prediction of gene regulatory binding sites using structure-based methods is useful for the inference of gene regulatory relationships in new species that are otherwise difficult to infer.

## Introduction

A large portion of cellular physiology and adaptation depends upon the finely tuned molecular interactions that constitute gene regulatory networks. Comparative and experimental analyses of the whole genomes of closely related organisms have recently revealed that a significant portion of the phenotypic diversity within and between species is due to changes in gene regulatory components [1], [2]. Indeed, while metabolic and signaling pathways vary little between related species, gene regulation varies significantly as a consequence of subtle genetic changes in gene regulatory network (GRN) architectures [3], [4]. A complete understanding of (and ability to predict) the consequences of genetically encoded regulatory variation in new species will require the ability to predict the consequences of this variation on transcription factor-DNA binding specificity.

The prediction of *cis-*regulatory binding sites of new transcription factors *in silico* will greatly facilitate the prediction of gene regulatory networks in new and understudied species. Sequence-based clustering and comparative genomics are successful at detecting specific classes of well-represented and well-studied transcription factor families [5], [6], but are often not comprehensive and do not take advantage of protein-DNA structural information. Structure-based methods to predict the DNA binding preferences of transcription factors have proven to accurately recapitulate the specificities of well studied regulatory proteins [7]–[11], providing an opportunity to make new predictions about gene regulation in diverse species that complement existing and orthogonal methods such as sequence-baesd *de novo* motif discovery. In this paper, protein-DNA structure-based prediction of sequence specificity [10]–[14] was used to predict the DNA sequence preferences, bindings sites and putative regulatory features of new and divergent archaeal feast/famine regulatory proteins in *Halobacterium salinarum* NRC-1. This serves as an example of *de novo* structure-based prediction of promoter binding sites for a relatively under-studied class of transcription factors.

Lrp-like feast/famine regulatory proteins (FFRPs) are widespread archaeal transcription factors that are closely related to the leucine regulatory protein (Lrp) and regulatory protein AsnC in proteobacteria [15]. They are generally amino-acid sensitive regulators of metabolism that can act upon either a few, or many genes. In *E. coli*, the Lrp regulator senses leucine and binds to and regulates over one hundred genes [16], whereas the closely related transcription factor AsnC senses asparagine and is only known to transcriptionally regulate a few genes, including itself (*asnC*) and asparagine synthetase (*asnA*) [17]. While only two genes encoding Lrp-like proteins exist in *E. coli* (*lrp* and *asnC*), numerous species of archaea contain multiple duplicated and diverged Lrp-like/FFRP genes in their genomes, which may reflect diverse metabolic modes and adaptations of these species to their environments [15]. The transcriptional regulation of large numbers genes by FFRPs, as in the case of *E. coli* Lrp, is a possible mechanism by which archaea are able to regulate their cellular physiology and metabolism over diverse and dynamic conditions in the environment.

The metabolite-dependent transcriptional activities of Lrp-like FFRPs depend on the nature of their C-terminal ‘Regulation of Amino acid Metabolism’ (RAM) domains, which bind amino acids and pyrimidines [18]. The binding of effector molecules by these RAM domains affects the multimeric states and DNA-binding properties of FFRP complexes [19], [20]. Much less is known about the DNA binding sites, gene regulatory mechanisms or transcriptional regulatory targets of FFRPs as compared to analogous transcriptional regulators of metabolism in proteobacteria, such as Lrp [16], FNR [21], and CRP [22]. High-order complexes between FFRPs and genomic DNA have been confirmed by electron microscopy [23], suggesting that FFRPs may exhibit nucleosome-like binding on the basis of macromolecular DNA flexibility. Transcriptional factors that regulate large numbers of genes (including Lrp, FNR and CRP) often exhibit relaxed sequence specificity, and this may be true for FFRPs as well. However, evidence suggests that both ‘indirect readout’ of macromolecular DNA flexibility and direct readout of specific promoter binding sites are important for their function [24], [25]. Considerable amino acid variation is evident in the DNA binding regions of the FFRPs (Fig. S1), likely leading to divergent DNA sequence preferences [24]. This includes putatively functional co-variation of the amino acids involved in protein-protein and protein-DNA interactions (Fig. S2). However, due to the novelty and complexity of the FFRP repertoires in archaea, the DNA-binding specificities of most FFRPs are unknown. The high sequence similarity of transcription factors in this family, and their preponderance for hetero-multimerization complicates sequence-based and experimental mapping efforts to elucidate their functional binding sites. We demonstrate the utility of a macromolecular structure-based prediction approach to overcome some of these challenges and to uncover novel insights into FFRP regulation in halophilic archaea.

## Materials and Methods

### Identification, comparison and sequence-based analysis of FFRP genes and proteins

The amino acid sequences of 101 FFRP DNA binding domains that bore similarity to the eight FFRP-like transcription factors in *H. salinarum* were collected using BLAST [26] and aligned using MUSCLE [27] and Jalview [28]. To detect the co-variation of amino acid identities between all pairs of aligned protein positions (*a, b*) in the DNA binding region of the FFRP family (Fig. S2), the mutual information (*MI*) metric was used as described in [29]:
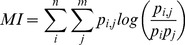
(1)where *n* and *m* represent all amino acid identities at alignment positions *a* and *b*, respectively, *p_i,j_* is the joint probability of two amino acid identities co-occuring in individual protiein sequences, and *p_j_* and *p_k_* and are the independent probabilities of the amino acids occuring at their respective positions. The protein sequence alignment, complete results, and the Python script used to compute mutual information are available online at: http://bragi.systemsbiology.net/data/FFRP/.

To identify conserved *cis*-regulatory sequences in the promoters of FFRP genes, orthologous genes in eighteen Halobacterial genomes [30], [31] that bore similarity to each FFRP gene in *H. salinarum* were identified by collecting reciprocal best BLASTp [26] matches between proteomes, with a minimum length of 50 amino acids, a minimum contiguous alignment coverage of 70% of the full-length query protein, and a minimum sequence protein identity of 50%. The promoter sequences upstream of each of their start positions were extracted from the corresponding genome sequences, and two non-coding *cis*-regulatory motifs were detected in these orthologous promoter regions using MEME [32] using the following arguments (all others default): -minw 10 -maxw 16.

### Structure-based prediction of transcription factor-DNA binding specificities

The amino acids in the DNA binding domains of each FFRP from *H. salinarum* were each separately threaded and structurally superimposed onto their analogous positions on the protein backbone of the crystal structure of the FL11 protein from *Pyrococcus horikoshii* [33] bound to DNA (pdb: 2e1c). All amino acid side-chains and nucleotide bases were energy minimized using the macromolecular modeling software Rosetta [34], which employs Monte Carlo methods that disretely search protein conformational space in order to minimize the estimated free energy of macromolecular complexes. In order to predict the DNA sequence preferences for each different FFRP, the identities of the nucleotide base pairs in the crystallographic DNA template were randomly sampled using a Monte Carlo simulated annealing procedure to find the lowest energy DNA sequence for a given FFRP model. Simultaneously, the amino acid side chain conformations of the protein were sampled, as well as the hydration states of each nucleotide base [10], [12]. A position weight matrix (PWM) was calculated for each FFRP model based on the relative frequency of nucleotide identities in an ensemble of the 100 lowest-energy DNA sequences. This protocol was used previously to recapitulate the DNA sequence preferences of a representative compendium of DNA-binding proteins with known DNA sequence specificities [12]. The scripts, parameters and protocols used to perform these simulations using the Rosetta software are available online at: http://bragi.systemsbiology.net/data/FFRP/. Predicted and experimentally measured PWMs were compared using TOMTOM [35] (using the default Pearson distance metric) between appropriate sets of measured and predicted PWMs in this paper, including: SELEX PWMs (*n_motifs_* = 4), PWMs discovered using MEME (*n* = 9), structurally predicted PWMs (*n* = 12), and PWMs iteratively refined to reflect putative genomic binding site sequences in *H. salinarum* (*n* = 8).

### Genome-wide binding site motif discovery, refinement and validation

Genome-wide FFRP-DNA binding sites were previously measured by chromatin immunoprecipitation and microarray hybridization (ChIP-chip) [36], similarly to previous experiments conducted in the same organism [37]. Briefly, in separate experiments for each FFRP, the TF was cloned and exogenously expressed from a cMyc-tagged vector (pMTF) during exponential and stationary phases of growth in normal growth media, proteins and DNA were crosslinked using formaldehyde, and FFRP-bound DNA was immunoprecipitated, sheared, amplified, labeled and hybridized to high density tiling arrays. An experimental negative control was also performed, using only the empty vector pMTF. These ChIP-chip data were processed using MeDiChI [38] to identify significant peaks under either exponential or stationary phase growth. Gene promoters in *Halobacterium salinarum* were considered binding targets if a ChIP-chip peak with a *p*-value less than 0.10 was present within 100 bp upstream of the transcriptional start site. For *de novo* discovery of genome-wide promoter DNA binding sites from ChIP experiments for each FFRP, MEME [32] was performed on the upstream non-coding promoter sequences of all genes with evidence of ChIP binding. The following parameters were used to run MEME: -minw 13, -maxw 17, -nmotifs 2, and MEME was supplied with a first-order background Markov model computed by over all input sequences. Upstream sequence regions tested for *de novo* motif detection included a range of possibilities, including −500 to +100 bp, −250 to 50 bp, and −100 to 0 bp relative to gene CDS starts or transcriptional start sites (In Fig. S3, the results for −100 to 0 bp are shown). *De novo* motif detection was also performed on the promoters of genes falsely identified as ‘bound’ in an experimental negative control experiment for ChIP-chip (empty vector; ‘pMTF’ in Fig. S3). For the purpose of inferring gene promoters which are bound by FFRPs, FIMO was used [32] to identify potential FFRP transcription factor binding sites (TFBS) in promoter regions from DNA-binding position weight matrices (PMWs) with a motif *p*-value below the default threshold (1×10^−4^). To adapt predicted PWMs to better reflect putative promoter binding site sequences as measured by ChIP experiments for each FFRP, an iterative procedure was used in which genomic sequences matching PWMs were found using FIMO with increasing stringency (*p*-value thresholds decreasing from 5×10^−4^ to 1×10^−4^) and used to update the starting position weight matrices. At each iteration, the motif PWM was updated by constructing a new PWM (**M**) from the empirically discovered TFBS and then mixing this with the seed motif according to the following expression with weight (*w*) increasing linearly with each iteration (*iter*) from 0.25 to 0.75:
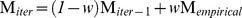
(2)


The R software routines used to perform this iterative motif refinement procedure are available online at: http://bragi.systemsbiology.net/data/FFRP/.

## Results and Discussion

### *De novo cis*-regulatory motif discovery is insufficient to explain FFRP DNA binding in *H. salinarum*

The *Halobacterium salinarum* NRC-1 genome encodes for at least eight transcription factors that possess a DNA-binding domain with sequence homology to FFRPs [30] (24–39% identity to FL11 protein from *Pyrococcus horikoshii* [33], 20–39% identity Lrp from *E. coli*). Considerable intra-species divergence in the DNA-binding domains of these putative FFRPs (Fig. S1) may reflect a diversification and evolution of the control of metabolism in *H. salinarum*. Understanding the impact of the molecular variation in this expanded transcription factor family on condition-dependent gene regulation represents a current challenge in microbial systems biology [16], [36], and this includes knowledge of how these transcription factors recognize the promoter sequences of the genes that they regulate.

Often it is possible to discover putative *cis*-regulatory transcription factor-DNA binding motifs through the *de novo* detection and analysis of enriched sequences in chromatin immunoprecipitation (ChIP) experiments for individual transcription factors [39]. For the FFRP transcription factors in *H. salinarum*, however, the detection of *cis*-regulatory motifs in ChIP-bound promoters did not yield PWMs that matched the experimentally measured PWMs for FFRPs in other species or clearly distinguished between FFRP-DNA binding site preferences (Fig. S3). The motifs discovered reproducibly by MEME in ChIP-bound promoter regions were AT-rich, TATA-like promoter elements that bore similarity to experimentally-determined SELEX PWMs for FFRPs in other archaeal species (*in vitro* SELEX; [24]) (Fig. S3D). While GC-rich DNA preferences have also been measured for certain FFRPs [24], [40], no patterns significantly matching these patterns were found using this approach. To further predict potential differences in the DNA sequence preferences of the eight different FFRPs in *H. salinarum*, we reasoned that an orthogonal protein-DNA structure-based approach to predict the sequence preferences of FFRPs could improve the differentiation between the DNA binding specificities of different FFRPs.

### Structure-based prediction of FFRP DNA-binding specificities

**Figure 1:**
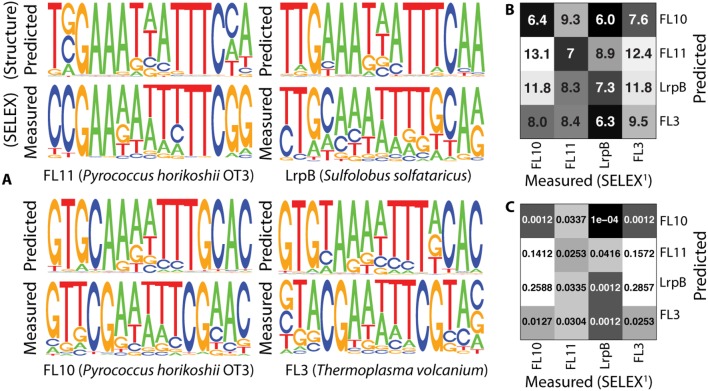
Comparison of predicted FFRP transcription factor DNA-binding specificities with *in vitro* (SELEX) measurements for four FFRPs. **A)** The predicted DNA-binding preferences of FFRPs FL11 from *P. horikoshii* (left) and LrpB from *S. solfataricus* (right) are highly similar to *in vitro* measurements of the specificities of these transcription factors. **B)** Euclidian distance matrix for DNA PWMs between each of the predicted and experimentally measured DNA-binding specificities. Dark boxes and low values and indicate higher similarity. **C)** The significance of similarities between PWMs in the context of comparison between all predicted and measured PWMs (mean reciprocal TOMTOM *p*-values, adjusted for multiple hypotheses with *n* = 16, Benjamini-Hochberg method).

The DNA-binding specificities of multiple FFRP transcription factors were predicted using a structure-based method that calculates the preferred DNA binding site sequences for a given transcription factor protein sequence [12]. For each FFRP protein sequence, its amino acids were substituted into corresponding positions the crystal structure of FL11 from *Pyrococcus horikoshii* OT3 [33], which is representative of the archaeal FFRP protein family. The optimal DNA sequence(s) for each model were then calculated on the basis of a physics-based energy force field and combined into a position weight matrix (PWM). These *In silico* structure-based predictions of DNA-binding sequence preferences significantly matched *in vitro* measurements [24] for multiple FFRP proteins (Figure 1), including FL10 and FL11 from *P. horikoshii* and LrpB from *Sulfolobus solfataricus*. The PWMs predicted for the FL11 and LrpB FFRPs significantly overlapped with experimentally determined profiles (*p*_adj_ = 0.0253 for FL11 and 0.0012 for LrpB) and correctly predicted the corresponding experimentally determined SELEX PWMs for these proteins [24] (Fig. 1B, 1C). The prediction for FL10 closely and significantly matched the SELEX PWM for this FFRP (*p* = 0.0012), but also predicted similarity to the SELEX PWM for LrpB (*p* = 0.00011). Similarly, the predicted PWM for the FL3 protein from *Thermoplasma volcanium*, while similar to the experimentally determined PWM (*p* = 0.0253), was also similar to the SELEX PWM for LrpB (*p* = 0.0012). While the predictive accuracy of the structure-based method was less than 100% over these test cases, the predictive value of this method was sufficient to investigate the hypothetically different DNA recognition specificities present within the complement of novel FFRP transcription factors in *Halobacterium salinarum*.

### Prediction of the DNA-binding specificities for all FFRPs in *Halobacterium salinarum*

**Figure 2:**
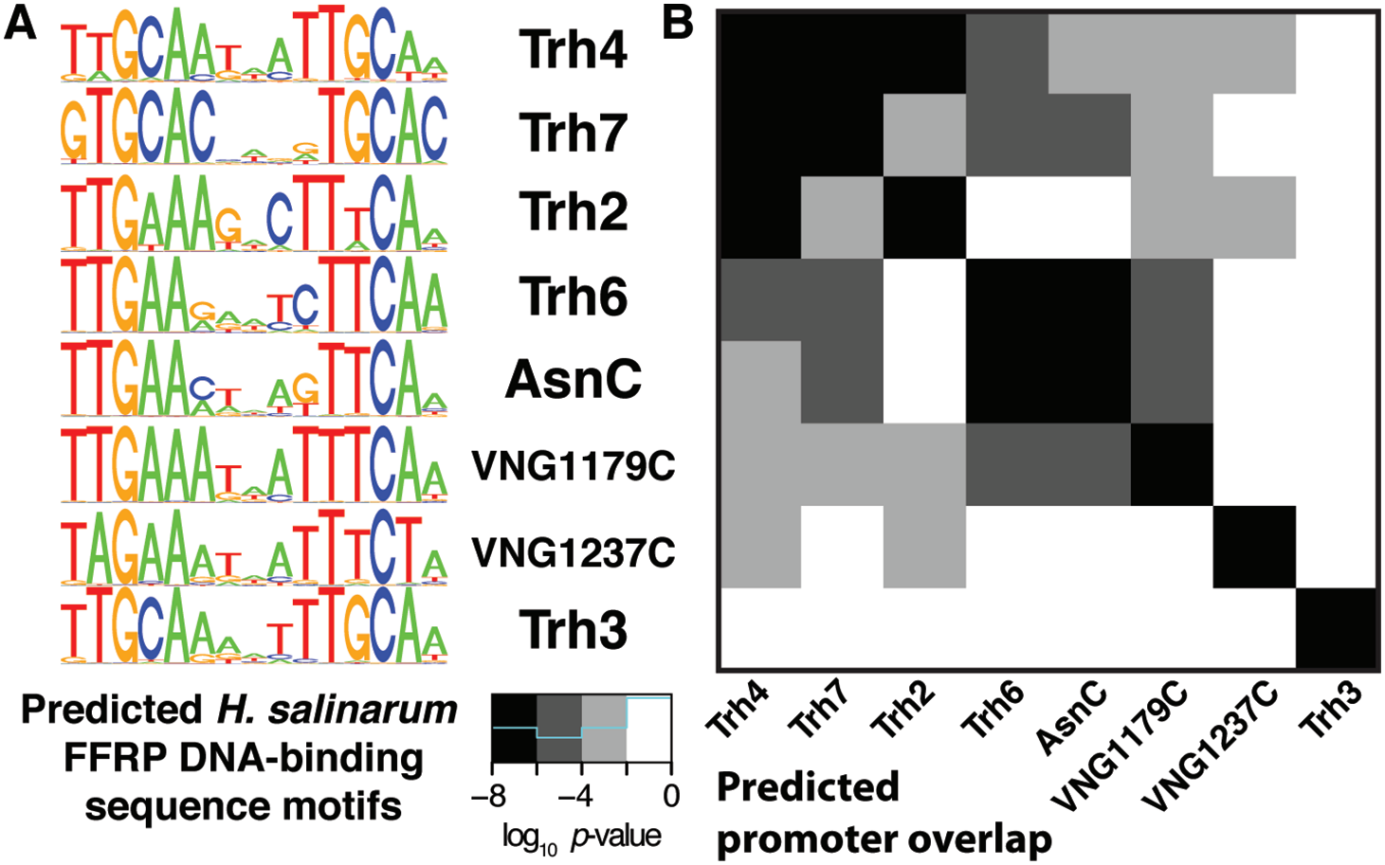
**A)** Predicted novel transcription factor-DNA binding site (TFBS) motifs for the FFRP repertoire in *Halobacterium salinarum* NRC-1. **B)** The statistical overlap between gene promoters that contain sequences matching one or more of the predicted FFRP DNA binding sites in (A). Dark boxes indicate higher overlap (hypergeometric *p*-value).

In order to further study the potential for divergent gene regulatory interactions for the FFRPs in *H. salinarum*, structure-based predictions of the DNA-binding specificities for each FFRP in this genome were produced using the crystal structure of FL11 from *P. horikoshii* as a structural template (Fig. 2). The resulting DNA-binding sequence motifs were distinct from previously determined binding site sequences for archaeal FFRPs [24] and able to partly differentiate between hypothetical FFRP DNA-binding specificities (Fig. 2, Fig. S4) on the basis of the protein sequence variation in their DNA binding domains. In addition, the structure-based predictions for FFRP specificities in *H. salinarum* bore higher similarity to the measured SELEX PWMs to FFRPs from other species than the PWMs obtained using MEME on ChIP-bound promoter regions (Fig. S4B). Predicted promoter binding targets based on these PWMs (Fig. 2B) were next compared to genome-wide binding measurements by chromatin immunoprecipitation (ChIP) and conserved DNA sequence motifs in the promoters of eighteen closely related halobacterial species.

### Prediction of FFRP *cis*-regulatory binding sites in *Halobacterium salinarum*

To infer transcription factor-binding sites (TFBS) for the FFRPs in *Halobacterium salinarum*, all gene promoters were searched using FIMO for significant occurrences of sequences similar to each predicted position weight matrix (PWM). This yielded between fourteen (Trh3) and forty-eight (Trh2) potentially regulated promoters per FFRP, with significant overlap in promoters with predicted binding sites (Fig. 2B). While several inferred regulatory targets matched ChIP-bound promoters for each FFRP, the overlap between predicted FFRP binding and genome-wide measurements by ChIP was generally insignificant (Table S1). The lack of high correspondence between predicted and measured genome-wide transcription factor binding site locations commonly occurs for transcription factors [41], and FFRPs in particular [25], [42]. Similarly for the FFRPs in *H. salinarum*, a lack of correspondence could occur due both to prediction and measurement errors, as well as biological effects including both inert and conditional binding [16]. It is also likely that FFRP binding to gene promoters is determined by more than simply the occurrence of unique DNA sequences, and is influenced by specific environmental contexts, protein-protein interactions, or the binding of their effector molecules, as suggested by recent investigations of the genome-wide binding patterns of other related Lrp-like transcription factors [43]. Notwithstanding these limitations, we next sought to improve the correspondence of predicted and measured promoter binding by refining the *de novo* predictions of DNA sequence preferences to reflect the occurrence of possible transcription factor binding site sequences in promoter regions of *H. salinarum* genes.

### Iterative refinement of DNA recognition sequences to reflect ChIP data

In order to improve the ability to distinguish between the putative target genes for different FFRPs, the PWMs were refined by adaptation to sequences occurring in the promoters of putatively regulated genes. For each FFRP PWM, matching binding site sequences in the promoters of ChIP-bound and/or co-expressed genes (significantly correlated in expression with the FFRP gene over many microarrays (up to *n_arrays_* = 1,495) with an empirical *p*-value< = 0.05) were converted into count matrices and mixed with predicted PWMs in an iterative fashion. This resulted in PWMs that better reflect actual promoter binding sites sequences in *H. salinarum*, and in several cases (AsnC, Trh2, Trh3, VNG1179C, VNG1237C) significantly enrich PWM hits in genes that are putatively co-regulated vs. all genes (Fig. S5, Fig. S6).

**Figure 3:**
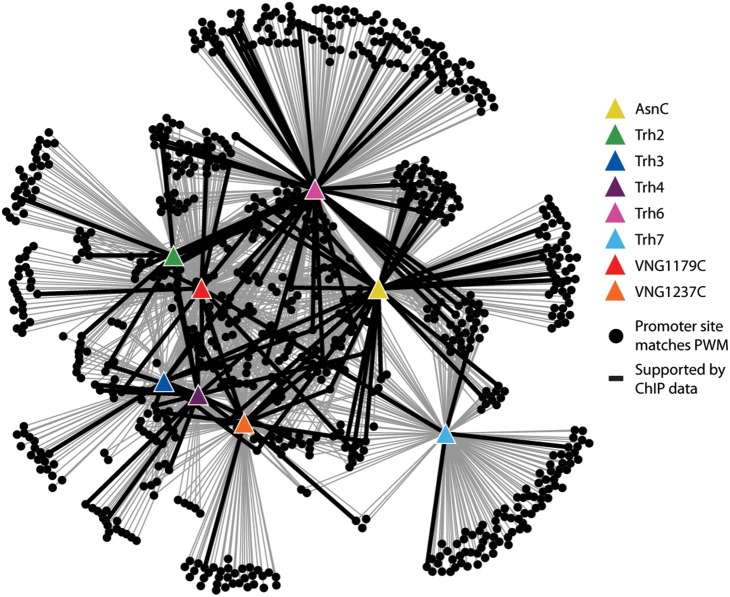
The set of inferred FFRP regulatory relationships in *H. salinarum* that is supported by structure-based prediction of FFRP transcription factor binding site preferences. Triangles are FFRP transcription factors and circles indicate genes whose promoters contain a sequence that matches a predicted PWM. Thick black lines indicate inferences that are supported by genome-wide binding measurements (ChIP).

Adaptation of the naively predicted PWMs to better reflect the sequences present in ChIP-bound promoters significantly improved the applicability of structure-based predictions to the *H. salinarum* genome (Fig. S5). The set of regulatory connections inferred here represent an improvement in our understanding of the gene regulatory architecture of *H. salinarum*. The inferred FFRP regulatory relationships supported by this analysis is broadly illustrated in Figure 3. This gene regulatory network includes likely gene regulatory influences by each FFRP based on shared FFRP *cis*-regulatory signals. Significant overlap between sets of predicted FFRP-regulated genes and experimental genome-wide binding measurements (ChIP) was observed for the PWMs predicted for Trh2 and Trh3 (Fig. S5, S6); in the cases of AsnC, VNG1179C, and VNG1237C, significant enrichment between predicted binding sites and significant ChIP binding was seen only among co-expressed genes.

The lack of overlap between the remaining majority of predicted and measured binding sites for all of the FFRPs (Fig. S6B) indicates the need for further refinement of the binding models, FFRP regulons, and genome-wide binding measurements. Measuring genome-wide binding using natively expressed FFRPs [44] under various conditions may be able to reconcile differences between genome wide binding measurements and PWM-based predictions. In addition, cooperation or competition between closely related FFRPs and other transcription factors may occur in promoters, complicating genome-wide binding patterns. The A-T rich (nAAn_{1–5}_TTn) motifs that are predicted binding sites for FFRPs in *H. salinarum* occur in many promoters throughout the genome, and overlap with core TATA elements that are bound by several related transcription factors, including multiple general transcription factors (GTFs) and TATA-binding proteins (TBPs) [31], [37], [45]. Thus while preferred nucleotide binding sequences can be inferred for the FFRPs, the full repertoire of their genome-wide binding locations and regulated genes may depend on indirect readout (nonlinear sequence-dependent macromolecular flexibility of the DNA molecule [46]), competitive or cooperative protein-protein interactions, metabolite binding, and condition-specific effects. In terms of expression, neither the ChIP-derived nor PWM-predicted regulons for any FFRP were significantly correlated in aggregate over all conditions, highlighting the need for improved modeling of the mechanisms by which FFRP binding relates to transcriptional regulation. Importantly, the roles of amino acid effector molecules in modulating FFRP binding and activity must be measured and incorporated into these networks.

Notwithstanding these limitations, the network inferred here contains predictions of new regulatory mechanisms for individual FFRPs. Below is an example of a strongly inferred gene regulatory relationship involving operon-like coordination of *carA* and *carB* genes in *H. salinarum* by Trh3.

### A Trh3 autoregulatory circuit coordinates non-operonic expression of the *carA* and *carB* genes

Interestingly, the prediction of Trh3 promoter binding sites infers that *H. salinarum* employs a divergent local *trans*-regulatory strategy to regulate genes (*carA* and *carB*) involved in amino acid metabolism, arginine and pyrimidine synthesis in response to amino acid levels, whereas *Escherichia coli* employs an operon [47]. The *carA* and *carB* genes in *Halobacterium salinarum* (VNG1815G and VNG1814G), which encode the carbamoyl phosphate synthetase small and large subunits, are adjacent to the *trh3* gene and are encoded on opposite strands of the chromosome, with distinctly independent promoter regions. This is in contrast to *E. coli*, in which *carA* and *carB* are joined into an operon that is regulated by ArgR and PurR repression [48].

**Figure 4:**
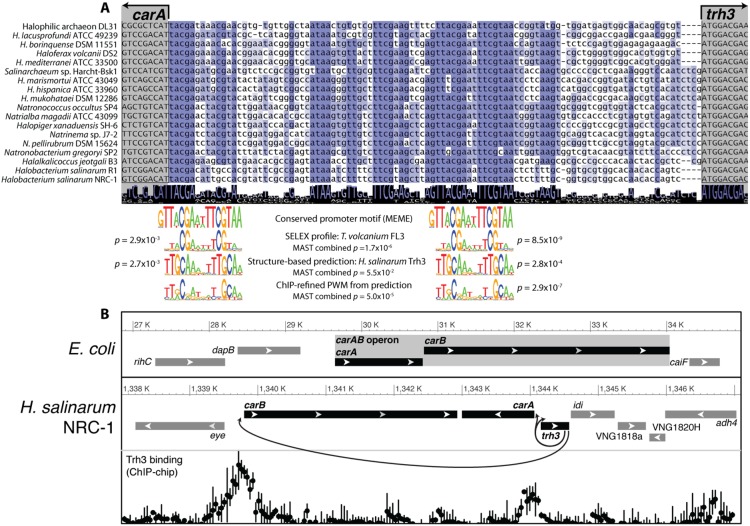
Prediction of *cis*-regulation by Trh3. **A)** Structure-based prediction of the Trh3 TFBS is similar to two DNA sequence regions in the shared *carA/trh3* promoter region that are conserved across eighteen halobacterial species. The experimentally measured TFBS of the Trh3 ortholog FL3 from *T. volcanium* also matches these conserved *cis*-regulatory sequences. **B)** In halobacterial species, the *trh3* gene product is a transcription factor that transcriptionally coordinates the expression of genomically separated subunits of carbamoyl phosphate synthetase (*carA* and *carB*), as well as itself.

In many bacterial species, transcription factors are often both autoregulatory (binding to their own promoters) and also regulate genes that are located in close proximity in the genome [5], [6]. Structure-based predictions of the DNA-binding specificity of the Trh3 transcription factor were the most similar to conserved *cis-*regulatory sequences in the shared promoter regions of both *carA* and *carB* in *H. salinarum*, as well as in the promoter region of its own gene, *trh3* (Fig. 4A, Fig. S4B Fig. S7). In fact, the *trh3* gene shares its promoter region with *carA*, and this region contains two distinct, conserved *cis*-regulatory signals that bear similarity to the predicted Trh3 binding motif (Fig. 4A). These signals are also matched by the *in vitro* specificity profile that was measured for the FFRP FL3 from *Thermoplasma volcanium*, which is the closest ortholog to the Trh3 protein for which *in vitro* measurements of DNA-binding specificity exist [24]. Binding of the Trh3 protein to the promoter regions of *trh3*, *carA* and *carB* was confirmed by chromatin immunoprecipitation (ChIP) experiments in *H. salinarum* (Fig. 4B).

**Figure 5:**
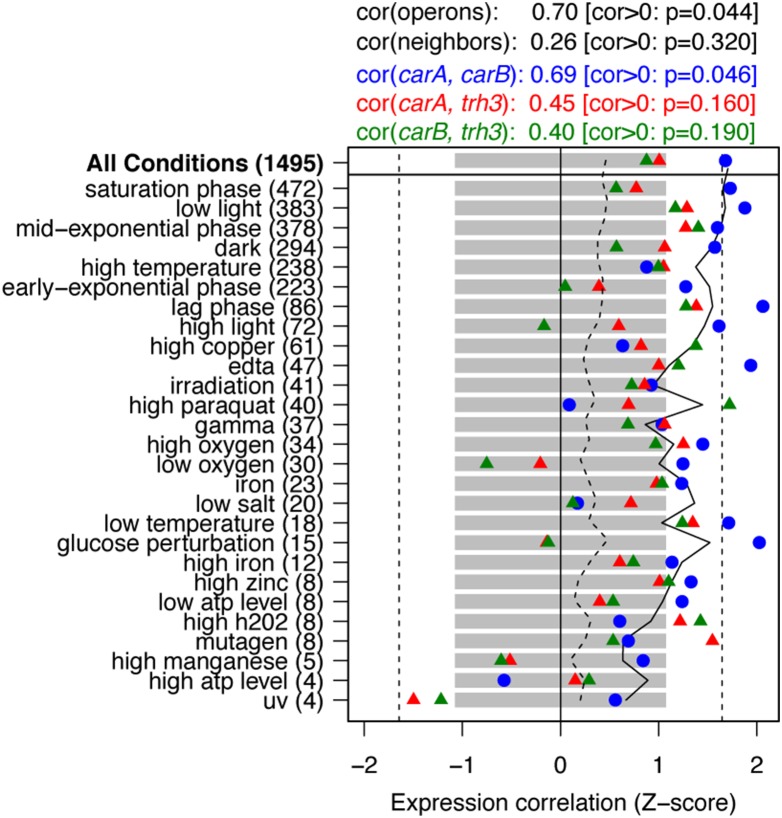
Gene expression correlations for *carA, carB,* and *trh3* over a large compendium of microarray experiments. The Z-scores (horizontal axis) for pairwise gene correlations are shown for several experimental conditions (left, numbers of arrays in parentheses). Gray bars represent +/−1 standard deviation over all conditional gene correlations; dashed lines indicate a *p*-value of 0.05. A solid black line indicates the average level of correlation between co-operonic genes, which is positive (*p*≤0.044). *carA* and *carB,* while non-operonic, are similarly correlated over all conditions (blue circles, *p*≤0.046). *carA* and *carB* are positively correlated with *trh3* (red and green triangles), but weakly so (*p*≤0.16 and 0.19, respectively), and not under all conditions. The mean correlation between neighboring, non-operonic genes (which includes the pairs *carA* vs. *carB* and *carA* vs. *trh3*) is also weakly positively correlated in general, but not significant (dashed black line; *p* = 0.32, *n* = 1,661 pairs of non-operonic neighbors).

To assess whether the regulation of *carA* and *carB* expression was maintained in operon-like coordination under the control of Trh3, genome-wide expression correlation values were compared for *trh3*, *carA*, *and carB* over hundreds of gene expression microarrays representing several distinct growth conditions (Fig. 5). The expression levels of *carA* and *carB* were highly correlated over the whole microarray compendium (*n*_arrays_ = 1,495; correlation coefficient: 0.69; *p* = 0.046), similar to *H. salinarum* genes within operons (correlation coefficient 0.70; *p* = 0.044). However, this correlation is not observed under certain stressful conditions (high temperature, high copper, high paraquat, low salt), due either to reduced or de-coupled transcription of these genes. The expression level of *trh3*, while positively correlated with *carA* and *carB*, was not alone adequate to explain the variation in expression of its targets (correlation coefficients: 0.45 and 0.40, *p* = 0.16 and 0.19, respectively). A missing factor to explain the regulatory activity of *trh3* on its targets is the level of its effector molecule, presumably arginine or lysine [36], [49], and the quantitative effect that this has on the activating or repressive functions of this transcription factor. Modulation by effector molecules is a crucial parameter to explain the activities of other Lrp-like proteins and FFRPs [15], [16], [49]. Nevertheless, the operon-like co-expression of *carA* and *carB*, together with evidence of site-specific *trh3* binding to their promoters suggests a novel, independent regulation of the *carA* and *carB* genes Trh3. The existence of this *trans*-regulatory mechanism in the genomes of archaea in place of the usual operon (as in *E. coli*) may be either an incidental outcome of genome evolution or the result of adaptive regulatory expansion and evolution in archaea.

## Conclusion

Understanding the novelty and complexity of gene regulatory networks in exotic and non-model organisms is challenging, due partly to the difficulty in predicting the DNA binding site preferences for expanded families of transcription factors (here, FFRPs in archaea). Using a structure-based modeling approach, we were able to predict the putative *cis*-regulatory binding sites and partial regulatory network for eight FFRPs in *H. salinarum*, which cannot be done using either knowledge from other bacteria, nor *de novo* motif discovery from genome-wide binding (ChIP) data. Contained within this *de novo*–predicted network are predictions of specific regulatory interactions (e.g. Trh3 binding and regulation of *carA* and *carB*) that are validated by experimental binding data and conserved across multiple closely related archaeal genomes. Thus the prediction of gene regulatory binding sites using *de novo* structure-based methods may be useful for the inference of gene regulatory relationships in new species that are difficult to infer by other means.

Supporting Information: supplementary files accompanying this manuscript including data, scripts and results are available online at: http://bragi.systemsbiology.net/data/FFRP/.

## Supporting Information

Figure S1 **Protein sequence alignment for FFRP DNA-binding regions in and several related species.**
(PNG)

Figure S2 **Highly co-variant amino acid positions in the FFRP-DNA binding domain.** The amino acid positions (31, 34, 37, 55, 56, 59, 60, 77, 79) of the FFRP DNA-binding domain that display the highest levels of mutual information with other amino acid identities in alignments of 101 homologous FFRP protein sequences are shown as space-filling spheres. Colors (cyan/blue, pink/purple) are for illustrative purposes to visualize the two distinct protein chains of the FL11 protein dimer. The oxygen and nitrogen atoms of the highlighted amino acid side chains are also colored red and blue, respectively. This figure is based on the structure of a dimer of FL11 DNA-binding domains from *Pyrococcus horikoshii* OT3 bound to DNA (pdb: 2e1c, Yokoyama et al. 2007 [33]).
(PNG)

Figure S3 **Enriched DNA sequence motifs discovered in ChIP-bound promoters using the MEME program.** The cis-regulatory motifs (**A**) detected in the promoters bound by plasmid-expressed FFRPs appear ambiguous and overlapping due to the prevalence of highly similar AT-rich and TATA core promoter elements found near measured binding site locations in hundreds of gene promoters. ‘pMTF’ indicates the results of *de novo* motif detection using MEME for the results of an experimental negative control (empty vector). **B**), the hypergeometric enrichment *p*-values for the occurrences of sequences matching each of these PWMs in the ChIP-bound promoters for each FFRP is shown. **C and D**) The significance of similarities between all MEME-derived PWMs (**C**) and all MEME and SELEX PWMs (**D**) according to TOMTOM (Pearson distance metric, *p*-value adjusted using the Benjamini-Hochberg method).
(PNG)

Figure S4 **Comparisons of predicted DNA binding PWMs for the FFRPs in** ***H. salinarum*** **to each other and to experimentally measured DNA binding PWMs for four FFRPs from different species.** In (**A**), numerical values are the significance of the similarities between PWMs according to TOMTOM are (Pearson distance metric, *p*-value adjusted using the Benjamini-Hochberg method). **B**) TOMTOM *p*-values for comparisons between all SELEX, MEME-derived, predicted, refined, and conserved PWMs (*carA, carB,* and *trh3*) presented in this manuscript.
(PNG)

Figure S5 **Progress of iterative refinement to improve the correspondence between** ***de novo*** **-predicted transcription factor binding site (TFBS) motifs and actual sequences occurring in experimentally-bound gene promoters, as measured by chromatin immunoprecipitation (ChIP).** ‘Correlated ChIP’ refers sets of genes that were both bound by the indicated FFRP according to ChIP, and also co-expressed. The *p-*value show on the vertical axis is the hypergeometric *p*-value for enrichment of promoters containing predicted binding sites in the set of experimentally bound vs. unbound promoters.
(PNG)

Figure S6 **DNA-binding preferences of** ***H. salinarum*** **FFRPs resulting from the refinement of structure-based predictions using ChIP and co-expression data.** A) Structure-based predictions of *H. salinarum* FFRP DNA-binding specificities. B) The PWMs in (A) were iteratively refined to reflect actual promoter binding site sequences for genes that were bound according to ChIP and co-expressed with the FFRP under at least one environmental condition. The ratio of bound and co-expressed genes with a detectable binding site sequence is shown at right, with hypergeometric *p*-values indicating the significance of this enrichment vs. all other promoters. Faded text (Trh4, Trh6, Trh7) indicates insignificant enrichment of the PWM in ChIP-bound and co-expressed genes.
(PNG)

Figure S7 **A conserved DNA sequence motif in the** ***carB*** **promoters of eighteen halobacterial species is similar to protein structure-based predictions of the DNA binding specificities of Trh3 from** ***Halobacterium salinarum*** **, as well as SELEX measurements for the orthologous FL3 protein from** ***T. volcanium*** **.**
(PNG)

Table S1 **The overlap of structurally predicted FFRP binding sites and chromatin immunoprecipitation (ChIP) experiments in** ***Halobacterium salinarum*** **.** Hypergeometric *p*-values (largely insignificant) are reported for the enrichment of promoters containing predicted binding sites in the set of experimentally bound vs. unbound promoters.
(DOCX)
