# Correction: Inference of Expanded Lrp-Like Feast/Famine Transcription Factor Targets in a Non-Model Organism Using Protein Structure-Based Prediction

**DOI:** 10.1371/journal.pone.0114450

**Published:** 2014-11-20

**Authors:** The PLOS ONE Staff

Two affiliations are missing for the fifth author. Nitin S. Baliga is affiliated with:

Institute for Systems Biology, Seattle, Washington, United States of America

Departments of Biology and Microbiology, University of Washington, Seattle, Washington, United States of America

Molecular and Cellular Biology Program, University of Washington, Seattle, Washington, United States of America

Lawrence Berkeley National Lab, Berkeley, California, United States of America
