# Supplementary figures and images for: Inference of Expanded Lrp-Like Feast/Famine Transcription Factor Targets in a Non-Model Organism Using Protein Structure-Based Prediction
Source: PLoS One. 2014 Sep 25;9(9):e107863. doi: 10.1371/journal.pone.0107863 (PMC4177876; doi:10.1371/journal.pone.0107863)

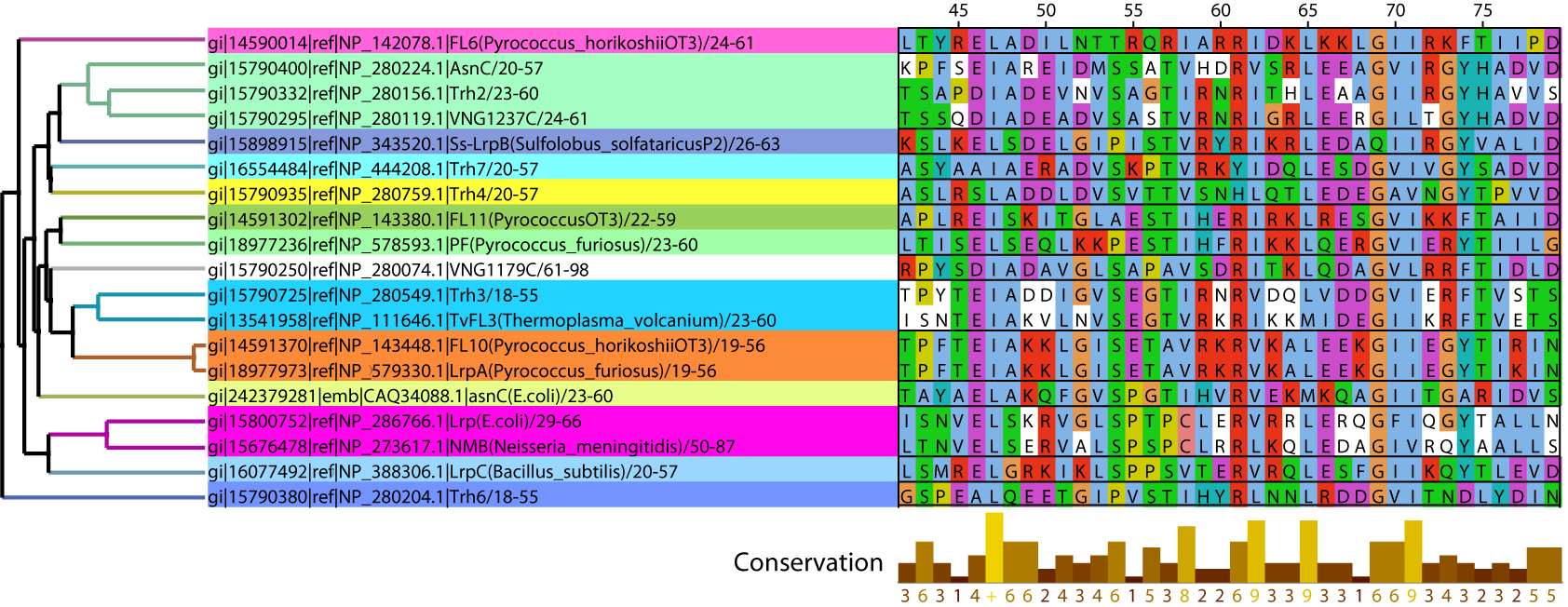

Supplement: Figure S1 — Protein sequence alignment for FFRP DNA-binding regions in and several related species. (PNG) [file pone.0107863.s001.png]

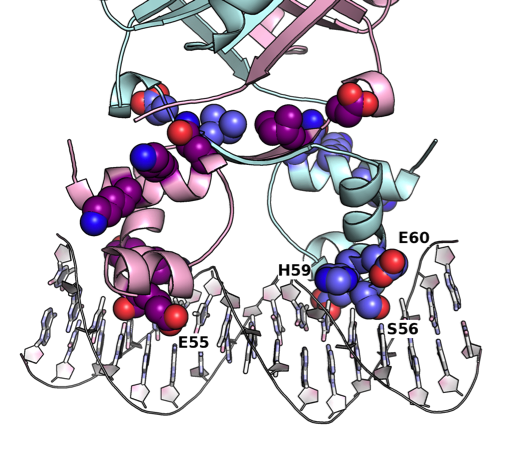

Supplement: Figure S2 — Highly co-variant amino acid positions in the FFRP-DNA binding domain. The amino acid positions (31, 34, 37, 55, 56, 59, 60, 77, 79) of the FFRP DNA-binding domain that display the highest levels of mutual information with other amino acid identities in alignments of 101 homologous FFRP protein sequences are shown as space-filling spheres. Colors (cyan/blue, pink/purple) are for illustrative purposes to visualize the two distinct protein chains of the FL11 protein dimer. The oxygen and nitrogen atoms of the highlighted amino acid side chains are also colored red and blue, respectively. This figure is based on the structure of a dimer of FL11 DNA-binding domains from Pyrococcus horikoshii OT3 bound to DNA (pdb: 2e1c, Yokoyama et al. 2007 [33]). (PNG) [file pone.0107863.s002.png]

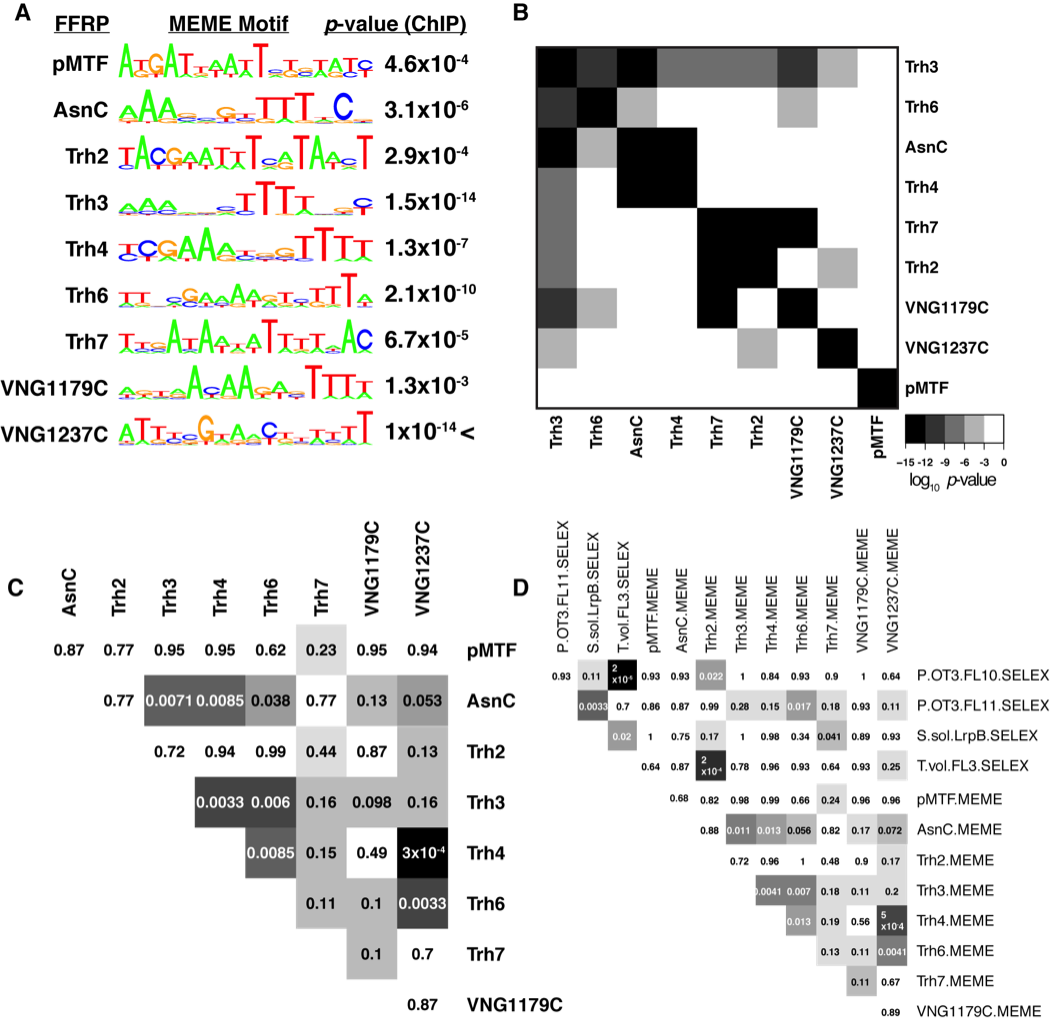

Supplement: Figure S3 — Enriched DNA sequence motifs discovered in ChIP-bound promoters using the MEME program. The cis-regulatory motifs (A) detected in the promoters bound by plasmid-expressed FFRPs appear ambiguous and overlapping due to the prevalence of highly similar AT-rich and TATA core promoter elements found near measured binding site locations in hundreds of gene promoters. ‘pMTF’ indicates the results of de novo motif detection using MEME for the results of an experimental negative control (empty vector). B), the hypergeometric enrichment p-values for the occurrences of sequences matching each of these PWMs in the ChIP-bound promoters for each FFRP is shown. C and D) The significance of similarities between all MEME-derived PWMs (C) and all MEME and SELEX PWMs (D) according to TOMTOM (Pearson distance metric, p-value adjusted using the Benjamini-Hochberg method). (PNG) [file pone.0107863.s003.png]

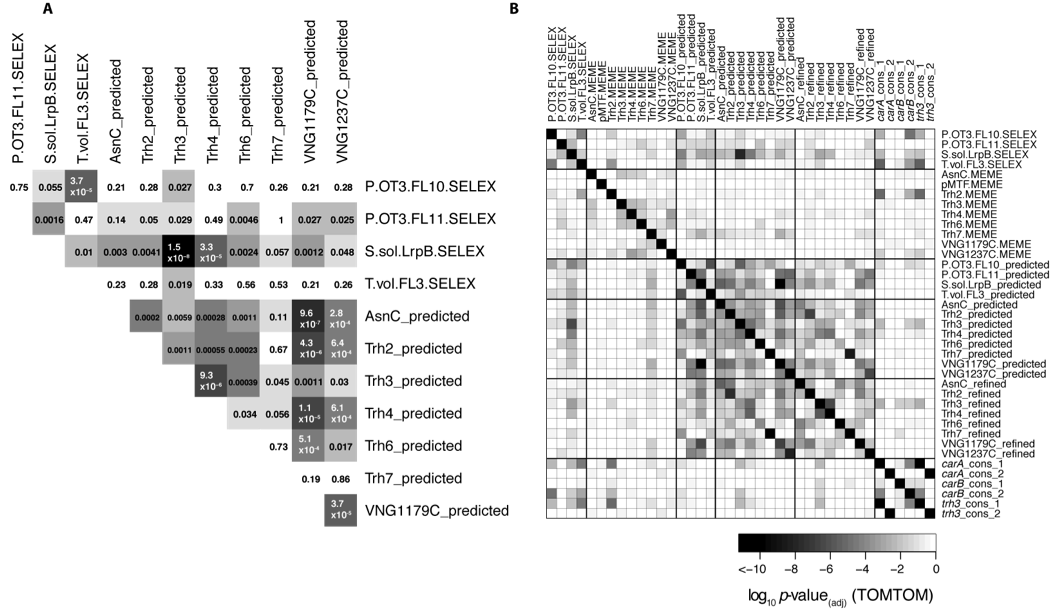

Supplement: Figure S4 — Comparisons of predicted DNA binding PWMs for the FFRPs in H. salinarum to each other and to experimentally measured DNA binding PWMs for four FFRPs from different species. In (A), numerical values are the significance of the similarities between PWMs according to TOMTOM are (Pearson distance metric, p-value adjusted using the Benjamini-Hochberg method). B) TOMTOM p-values for comparisons between all SELEX, MEME-derived, predicted, refined, and conserved PWMs (carA, carB, and trh3) presented in this manuscript. (PNG) [file pone.0107863.s004.png]

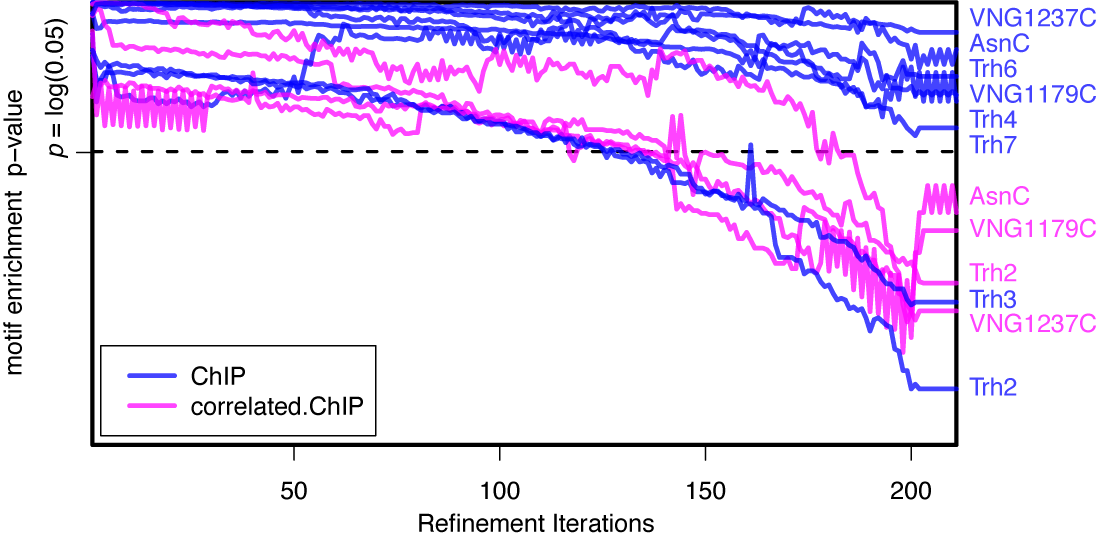

Supplement: Figure S5 — Progress of iterative refinement to improve the correspondence between de novo -predicted transcription factor binding site (TFBS) motifs and actual sequences occurring in experimentally-bound gene promoters, as measured by chromatin immunoprecipitation (ChIP). ‘Correlated ChIP’ refers sets of genes that were both bound by the indicated FFRP according to ChIP, and also co-expressed. The p-value show on the vertical axis is the hypergeometric p-value for enrichment of promoters containing predicted binding sites in the set of experimentally bound vs. unbound promoters. (PNG) [file pone.0107863.s005.png]

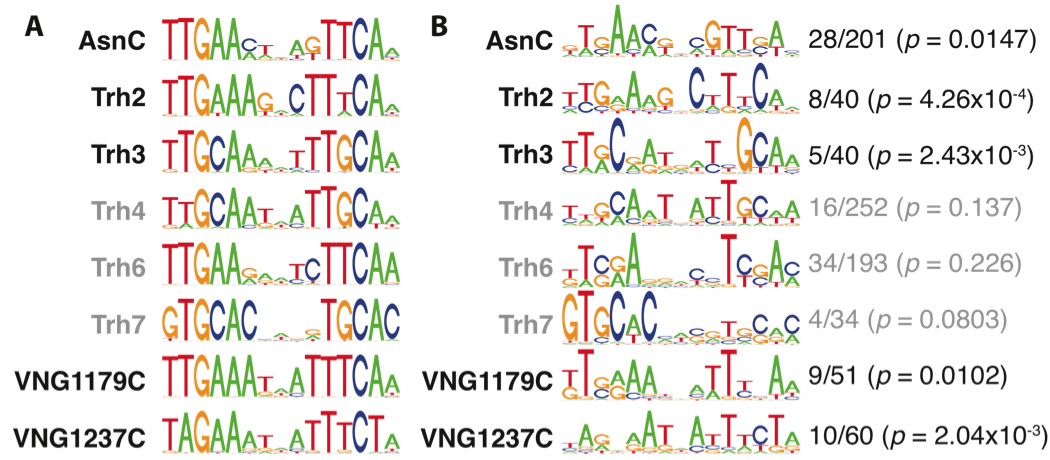

Supplement: Figure S6 — DNA-binding preferences of H. salinarum FFRPs resulting from the refinement of structure-based predictions using ChIP and co-expression data. A) Structure-based predictions of H. salinarum FFRP DNA-binding specificities. B) The PWMs in (A) were iteratively refined to reflect actual promoter binding site sequences for genes that were bound according to ChIP and co-expressed with the FFRP under at least one environmental condition. The ratio of bound and co-expressed genes with a detectable binding site sequence is shown at right, with hypergeometric p-values indicating the significance of this enrichment vs. all other promoters. Faded text (Trh4, Trh6, Trh7) indicates insignificant enrichment of the PWM in ChIP-bound and co-expressed genes. (PNG) [file pone.0107863.s006.png]

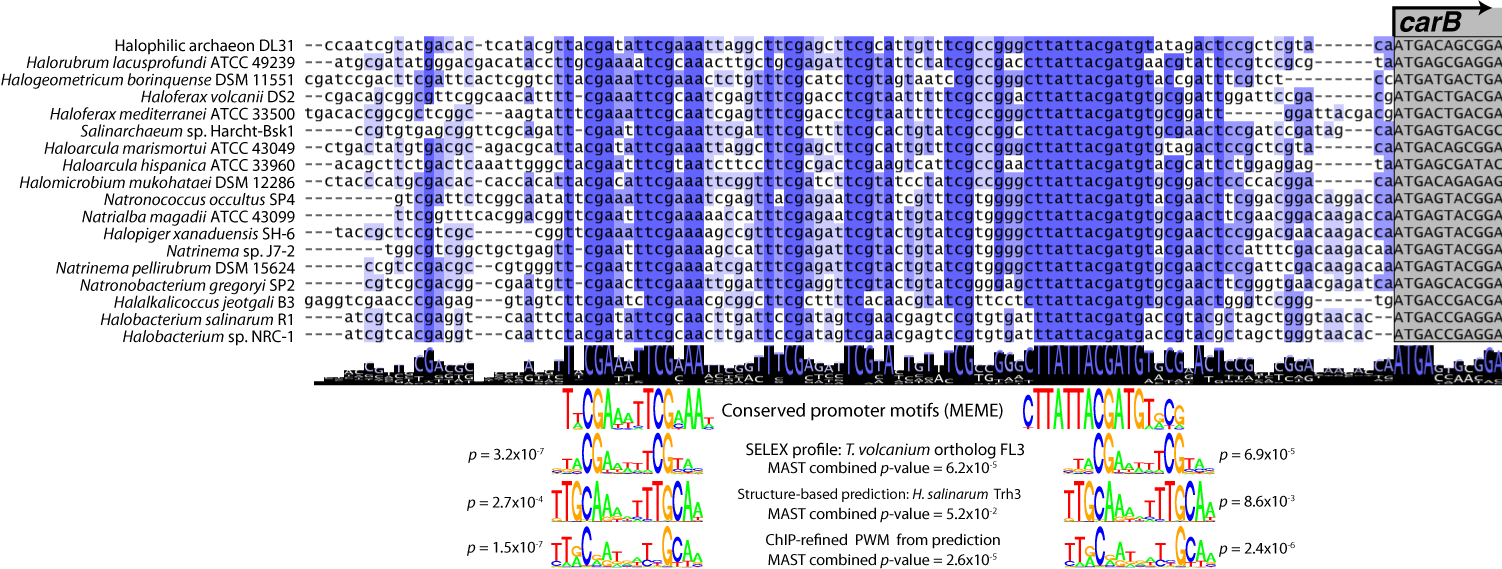

Supplement: Figure S7 — A conserved DNA sequence motif in the carB promoters of eighteen halobacterial species is similar to protein structure-based predictions of the DNA binding specificities of Trh3 from Halobacterium salinarum , as well as SELEX measurements for the orthologous FL3 protein from T. volcanium . (PNG) [file pone.0107863.s007.png]
