# Supplementary material for: Inference of Expanded Lrp-Like Feast/Famine Transcription Factor Targets in a Non-Model Organism Using Protein Structure-Based Prediction
Source: PLoS One. 2014 Sep 25;9(9):e107863. doi: 10.1371/journal.pone.0107863 (PMC4177876; doi:10.1371/journal.pone.0107863)
Supplement: Table S1 — The overlap of structurally predicted FFRP binding sites and chromatin immunoprecipitation (ChIP) experiments in Halobacterium salinarum . Hypergeometric p-values (largely insignificant) are reported for the enrichment of promoters containing predicted binding sites in the set of experimentally bound vs. unbound promoters. (DOCX) [file pone.0107863.s008.docx]

**Table S1.** The overlap of structurally predicted FFRP binding sites and chromatin immunoprecipitation (ChIP) experiments in *Halobacterium salinarum*. Hypergeometric *p*-values (largely insignificant) are reported for the enrichment of promoters containing predicted binding sites in the set of experimentally bound vs. unbound promoters.

| **FFRP** | **ChIP-bound genes with predicted FFRP binding sites** | **Correspondence over all ChIP data (Hypergeometric *p*-value)** |
| --- | --- | --- |
| AsnC | VNG0110C, VNG0255C, VNG0181G, VNG0689G, VNG1467G, VNG2552G, VNG0213H, VNG1910H, VNG2656H, VNG0615C, VNG0180G, VNG2649G, VNG2174H, VNG6378H | 0.65 |
| Trh2 | VNG2620G, VNG0966G, VNG6201G | 0.97 |
| Trh3 | VNG0149G, VNG1816G, VNG1214H, VNG1815G, VNG1096H | 0.021 |
| Trh4 | VNG2451H, VNG6143H | 0.97 |
| Trh6 | VNG0303G, VNG0966G | 0.99 |
| Trh7 | VNG1838H, VNG0994H, VNG1839H, VNG6391H | 0.35 |
| VNG1179C | VNG2620G, VNG1977H, VNG2116C, VNG0966G, VNG0994H, VNG6306C, VNG6182H | 0.99 |
| VNG1237C | VNG1816G, VNG0068H, VNG0657G, VNG1815G, VNG0994H, VNG2174H | 0.76 |
